# Supplementary material for: The ELAVL3/MYCN positive feedback loop provides a therapeutic target for neuroendocrine prostate cancer
Source: Nat Commun. 2023 Nov 28;14:7794. doi: 10.1038/s41467-023-43676-3 (PMC10684895; doi:10.1038/s41467-023-43676-3)
Supplement: Supplementary file 1 — Supplementary Information [file 41467_2023_43676_MOESM1_ESM.pdf]

SUPPLEMENTARY INFORMATION

The ELAVL3/MYCN positive feedback loop provides a therapeutic target for neuroendocrine prostate cancer

Authors:

Yiyi Ji[1], Weiwei Zhang[1], Kai Shen[1], Ruopeng Su[1], Xinyu Liu[1], Zehua Ma[1], Bo Liu[1], Cong Hu[1], Yizheng Xue[1], Zhixiang Xin[1], Yi Yang[1], Ang Li[1], Zhou Jiang[2], Na Jing[3], Helen He Zhu[3], Liang Dong[1], Yinjie Zhu[1], Baijun Dong[1], Jiahua Pan[1], Qi Wang[1,4*], Wei Xue[1*]

These authors contributed equally: Yiyi Ji, Weiwei Zhang, and Kai Shen.

Affiliations:

1. Department of Urology, Ren Ji Hospital, Shanghai Jiao Tong University School of Medicine, Shanghai 200120, China.

2. Department of Pathology, Ren Ji Hospital, Shanghai Jiao Tong University School of Medicine, Shanghai 200120, China.

3. State Key Laboratory of Oncogenes and Related Genes, Ren Ji Med-X Stem Cell Research Center, Shanghai Cancer Institute & Department of Urology, Ren Ji Hospital, Shanghai Jiao Tong University School of Medicine, Shanghai, China.

4. Shanghai Key Laboratory for Tumor Microenvironment and Inflammation, School of Medicine, Shanghai Jiao Tong University, Shanghai 200120, China.

*** Corresponding author:**

Qi Wang, Department of Urology, Ren Ji Hospital, Shanghai Jiao Tong University School of Medicine, Shanghai 200120, China; Phone: 86-21-63846590; Fax: 86-21-58394262; E-mail: wqi@sjtu.edu.cn

Wei Xue, Department of Urology, Ren Ji Hospital, Shanghai Jiao Tong University School of Medicine, Shanghai 200120, China; Phone: 86-21-63846590; Fax: 86-21-58394262; E-mail: xuewei@renji.com.

Supplementary Figures S1-S8

Supplementary Table 1-6

Supplementary Figure S1

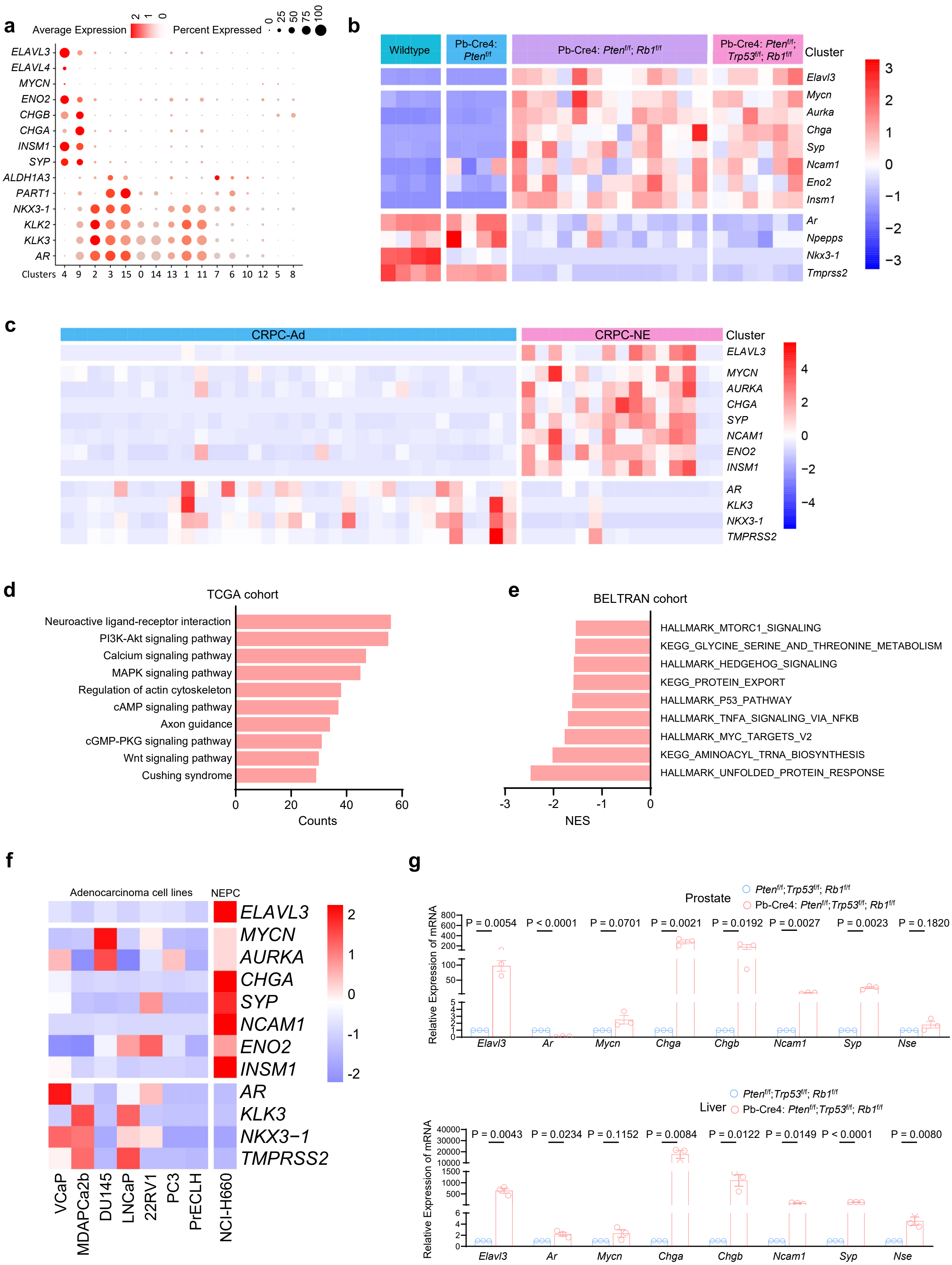

Supplementary Figure S1. ELAVL3 is associated with neuroendocrine differentiation of prostate cancer.

a, Dot plots illustrating the expression level of *ELAVL3*, *ELAVL4*, *MYCN*, neuroendocrine-related genes, and AR-related genes across 16 single-cell clusters. **b**, Heatmap showing the expression of *Elavl3*, NE-related genes, and Ar-related genes from GSE90891[1] (n = 4 wildtype samples, n = 4 Pb-Cre4: *Pten*$^{f/f}$ samples, n = 13 Pb-Cre4: *Pten*$^{f/f}$; *Rb1*$^{f/f}$ samples, n = 6 Pb-Cre4: *Pten*$^{f/f}$; *Trp53*$^{f/f}$; *Rb1*$^{f/f}$ samples). **c**, Heatmap showing the expression of *ELAVL3*, NE-related genes, and AR-related genes from the Beltran 2016 cohort[2] (n = 34 CRPC-Ad samples, n = 15 CRPC-NE samples). **d**, KEGG pathway enrichment analysis of samples with high ELAVL3 expression (top 50%) versus low ELAVL3 expression (bottom 50%) from the TCGA cohort[3]. **e**, KEGG pathway enrichment analysis of samples with high ELAVL3 expression (top 50%) versus low ELAVL3 expression (bottom 50%) from the Beltran 2016 cohort[2]. **f**, Heatmap showing the expression of *ELAVL3*, NE-related genes, and AR-related genes in prostate cell lines from the Cancer Cell Line Encyclopedia. **g**, QPCR showing relative expression of *Elavl3*, *Ar*, and NE-related genes in primary tumors (bottom) and liver metastasis samples (upper) from mice groups: *Pten*$^{f/f}$; *Trp53*$^{f/f}$; *Rb1*$^{f/f}$ (n = 3) and Pb-Cre4: *Pten*$^{f/f}$; *Trp53*$^{f/f}$; *Rb1*$^{f/f}$ (n = 3); each dot represents tissue from an individual mouse. Data presented as mean ± s.e.m. (**g**). Statistical significance was determined by two-tailed unpaired Student's t-test (**g**). CRPC-NE, castration resistant prostate cancer-neuroendocrine differentiation. CRPC-Ad, castration resistant prostate cancer-adenocarcinoma. Source data are provided as a Source Data file.

Supplementary Figure S2

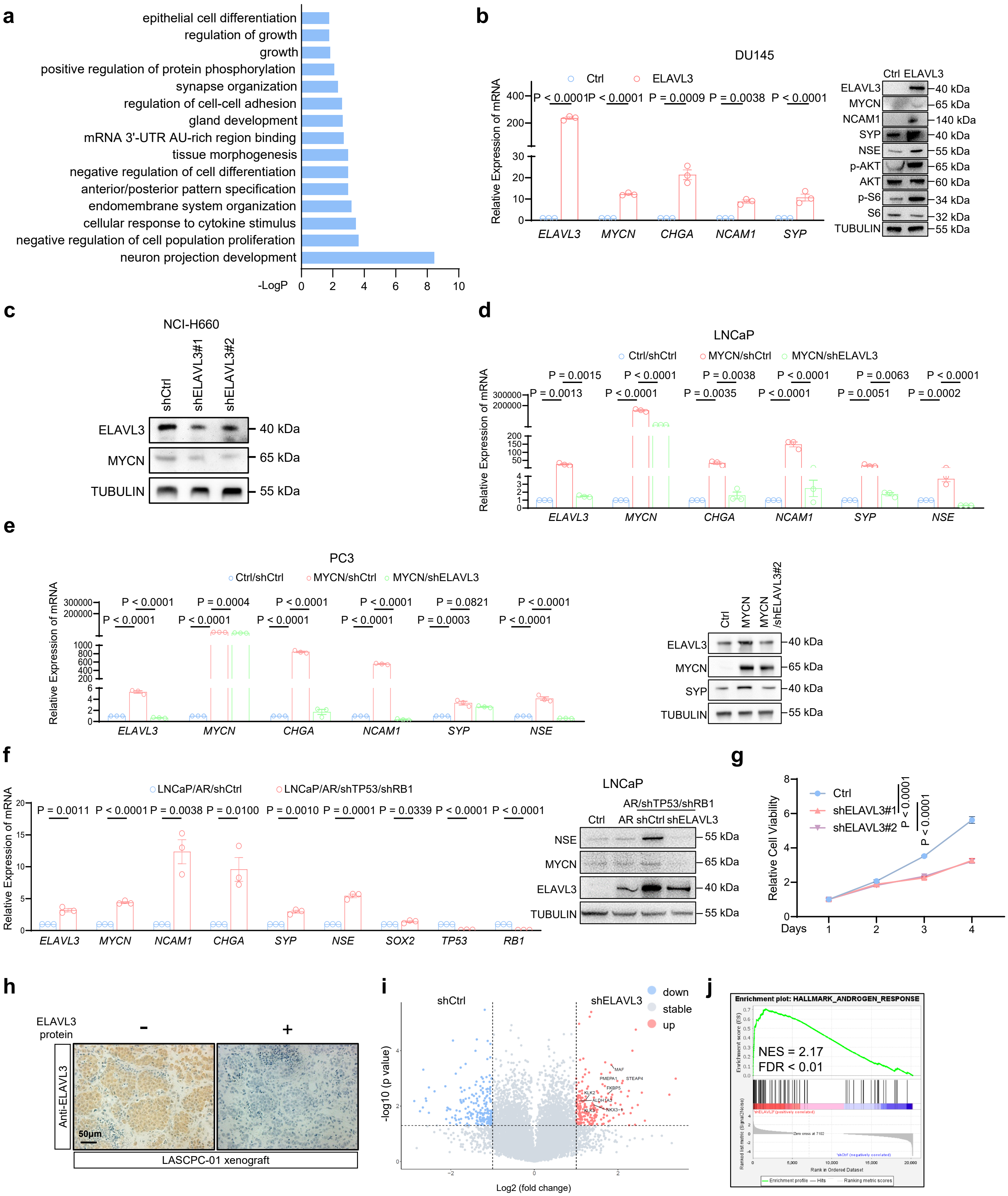

Supplementary Figure S2. ELAVL3 is essential for the development and maintenance of neuroendocrine prostate cancer

a, Gene Ontology enrichment analysis of samples with transcripts bound to ELAVL3-V5 versus IgG from PC3/ELAVL3-V5 or DU145/ELAVL3-V5. **b**, QPCR showing relative mRNA expression of *ELAVL3* and NE-related genes (*MYCN*, *NCAM1*, and *SYP*) in DU145/Ctrl and DU145/ELAVL3 (left). Western blot showing protein expression of ELAVL3, NE-related genes (MYCN, NCAM1, SYP, and NSE), p-AKT/AKT, and p-S6/S6 in DU145/Ctrl and DU145/ELAVL3 cells (right). **c**, Western blot showing expression of ELAVL3 and MYCN in indicated NCI-H660 cells. **d**, QPCR showing relative mRNA

expression of *ELAVL3* and NE-related genes (*MYCN*, *CHGA*, *NCAM1*, *SYP*, and *NSE*) in indicated LNCaP cells. **e**, QPCR showing relative mRNA expression of *ELAVL3* and NE-related genes (*MYCN*, *CHGA*, *NCAM1*, *SYP*, and *NSE*) in indicated PC3 cells (left, n = 3 biologically independent experiments). Western blot showing expression of ELAVL3, NE-related genes (MYCN and SYP) in indicated PC3 cells (right). **f**, QPCR showing relative mRNA expression of ELAVL3 and NE-related genes (*MYCN*, *NCAM1*, *CHGA*, *SYP*, and *NSE*) in LNCaP/AR/shCtrl and LNCaP/AR/shTP53/shRB1 (left). Western blot showing protein expression of ELAVL3, NE-related genes (NSE and MYCN) in indicated LNCaP cells (right). **g**, Cell viability of indicated LASCPC-01 cells at different time points (n = 3 biologically independent experiments). **h**, Representative immunohistochemistry staining of ELAVL3 in LASCPC-01 xenografts with/without recombinant ELAVL3 protein incubation (scale bar, 50 μm). **i**, Volcano plots showing differentially expressed genes in shELAVL3 versus shCtrl of LNCaP/AR/shTP53/shRB1 cells. Red or blue dots, fold change > 2; adjusted P-value < 0.05. RNA-seq was performed on three independent samples. **j**, Gene set enrichment analysis of differentially expressed genes using hallmark gene sets in shELAVL3 versus shCtrl of LNCaP/AR/shTP53/shRB1 cells. RNA-seq was performed on three independent samples. Data presented as mean ± s.e.m. (**b,d,e,f,g**). Statistical significance was determined by one-way ANOVA with Dunnett's multiple comparisons (**d,e,g**), or two-tailed unpaired Student's t-test (**b,f**). Western blot experiments were repeated three times independently, with similar results (**b,c,e,f**). QPCR experiments were conducted n = 3 biologically independent experiments. Source data are provided as a Source Data file.

Supplementary Figure S3

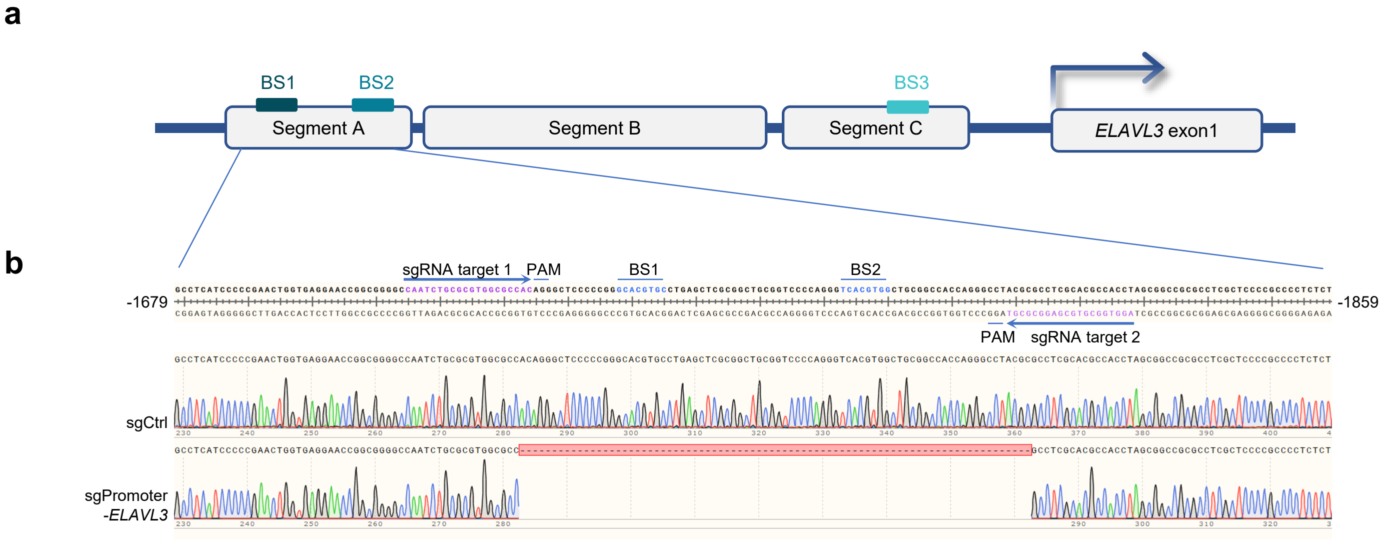

Supplementary Figure S3. MYCN transcriptionally upregulates ELAVL3 expression

a, Schematic diagram showing putative MYCN binding motifs on *ELAVL3* promoter and segments designed for luciferase reporter assay. **b**, Sequencing analysis of genomic DNA from HEK293T/sgCtrl and single-cell clone of HEK293T/sgELAVL3-promoter cells with two knock-out sgRNAs. The blue line with/without an arrow indicates the sgRNA-targeting sequence and PAM, respectively. The deletion mutation sequence is indicated by a solid red box.

Supplementary Figure S4

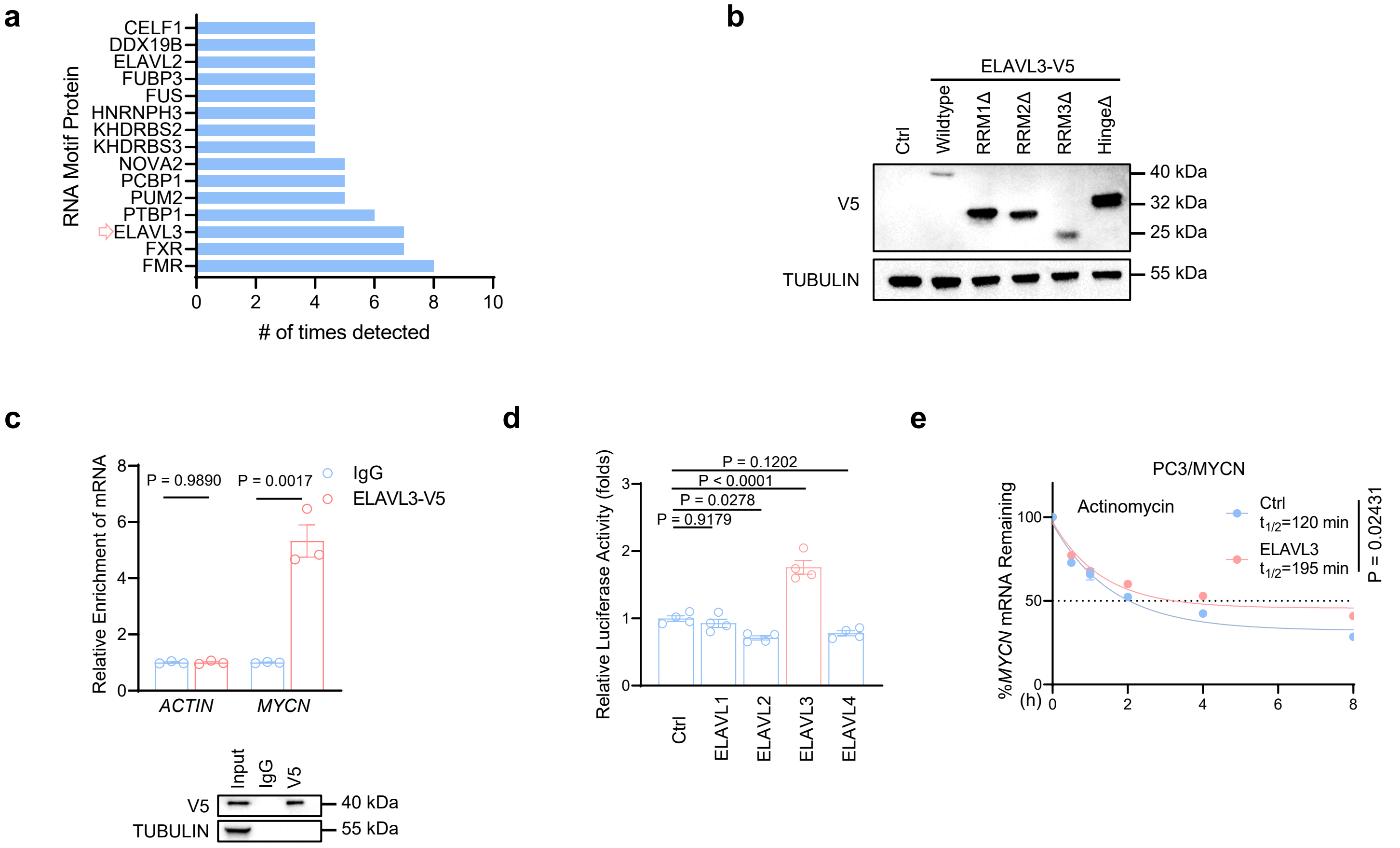

Supplementary Figure S4. ELAVL3 binds and stabilizes the mRNA of MYCN

a, Putative RNA-binding proteins within *MYCN* mRNA 3'-UTR provided by catRAPID. **b**, Western blot showing wildtype and indicated motif-deletion of ELAVL3-V5 protein in PC3 cells. **c**, Ribonucleoprotein immunoprecipitation (RIP) assay using either V5 antibody or IgG showing the relative enrichment of *MYCN* mRNA in LNCaP/ELAVL3 (upper, n = 3 biologically independent experiments). Western blot showing ELAVL3 was pulled down by V5 antibody (bottom). **d**, Relative luciferase activities driven by full-length of *MYCN* mRNA 3'-UTR reporters in HEK293T cells with ELAV family (n = 3 independently transfected replicates). **e**, *MYCN* mRNA decay in PC3/MYCN with/without ELAVL3 expression at indicated time points following 5 µg/mL Actinomycin D treatment (n = 3 biologically independent experiments). Data presented as mean ± s.e.m. (**c,d,e**). Statistical significance was determined by two-tailed unpaired Student's t-test (**c,e**), or one-way ANOVA with Sidak's multiple comparisons (**d**). Western blot experiments were repeated three times independently, with similar results (**b,c**). Source data are provided as a Source Data file.

Supplementary Figure S5

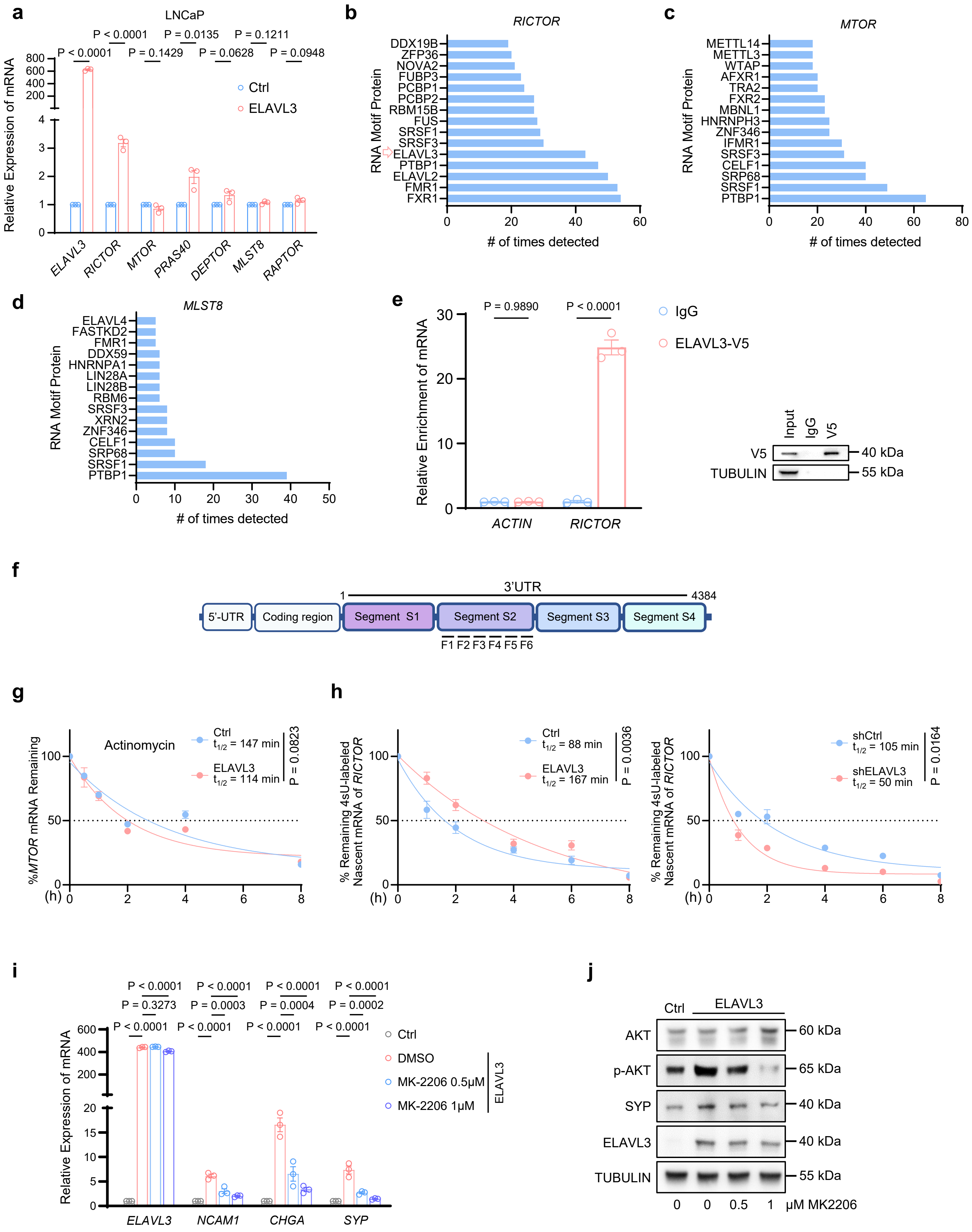

Supplementary Figure S5. ELAVL3 binds and stabilizes the mRNA of RICTOR

a, QPCR showing relative mRNA expression of *ELAVL3*, mTORC2-related genes (*RAPTOR*, *MTOR*, *RICTOR*, *MLST8*, *PRAS40*, and *DEPTOR*) in LNCaP/Ctrl and LNCaP/ELAVL3 (n = 3 biologically independent experiments). **b**, Putative RNA-binding proteins within *RICTOR* mRNA 3'-UTR provided by

catRAPID. **c**, Putative RNA-binding proteins within *MTOR* mRNA 3'-UTR provided by catRAPID. **d**, Putative RNA-binding proteins within *MLST8* mRNA 3'-UTR provided by catRAPID. **e**, RIP assay using either V5 antibody or IgG showing the relative enrichment of *RICTOR* mRNA in LNCaP/ELAVL3 cells (upper, n = 3 biologically independent experiments). Western blot showing that ELAVL3 was pulled down by V5 antibody (bottom). **f**, Schematic diagram showing putative ELAVL3 binding sites on *RICTOR* mRNA 3'-UTR, segments designed for luciferase reporter assay, and fragments (F1-F6) designed for RNA pull-down assays. **g**, *MTOR* mRNA decay in LASCPC-01/Ctrl and LASCPC-01/ELAVL3 at indicated time points post-treatment with 5 µg/mL Actinomycin D (n = 3 biologically independent experiments). **h**, RNA decay of 4sU-labeled nascent *RICTOR* in LASCPC-01/Ctrl and LASCPC-01/ELAVL3 cells at indicated time points (left). 4sU-labeled nascent *RICTOR* mRNA decay in LASCPC-01/shCtrl and LASCPC-01/shELAVL3 cells at indicated time points (n = 3 biologically independent experiments). **i**, QPCR showing the level of *ELAVL3* and NE-related genes (*NCAM1*, *CHGA*, and *SYP*) in PC3/Ctrl and PC3/ELAVL3 treated with MK-2206. **j**, Western blot showing the level of ELAVL3, SYP, p-AKT, and AKT in PC3/Ctrl and PC3/ELAVL3 cells treated with indicated MK-2206 (n = 3 biologically independent experiments). Data presented as mean ± s.e.m. (**a,e,g,h,i**). Statistical significance was determined by two-tailed unpaired Student's t-test (**a,e,g,h**), or one-way ANOVA with Dunnett's multiple comparisons (**i**). Western blot experiments were repeated three times independently, with similar results (**e,j**). Source data are provided as a Source Data file.

Supplementary Figure S6

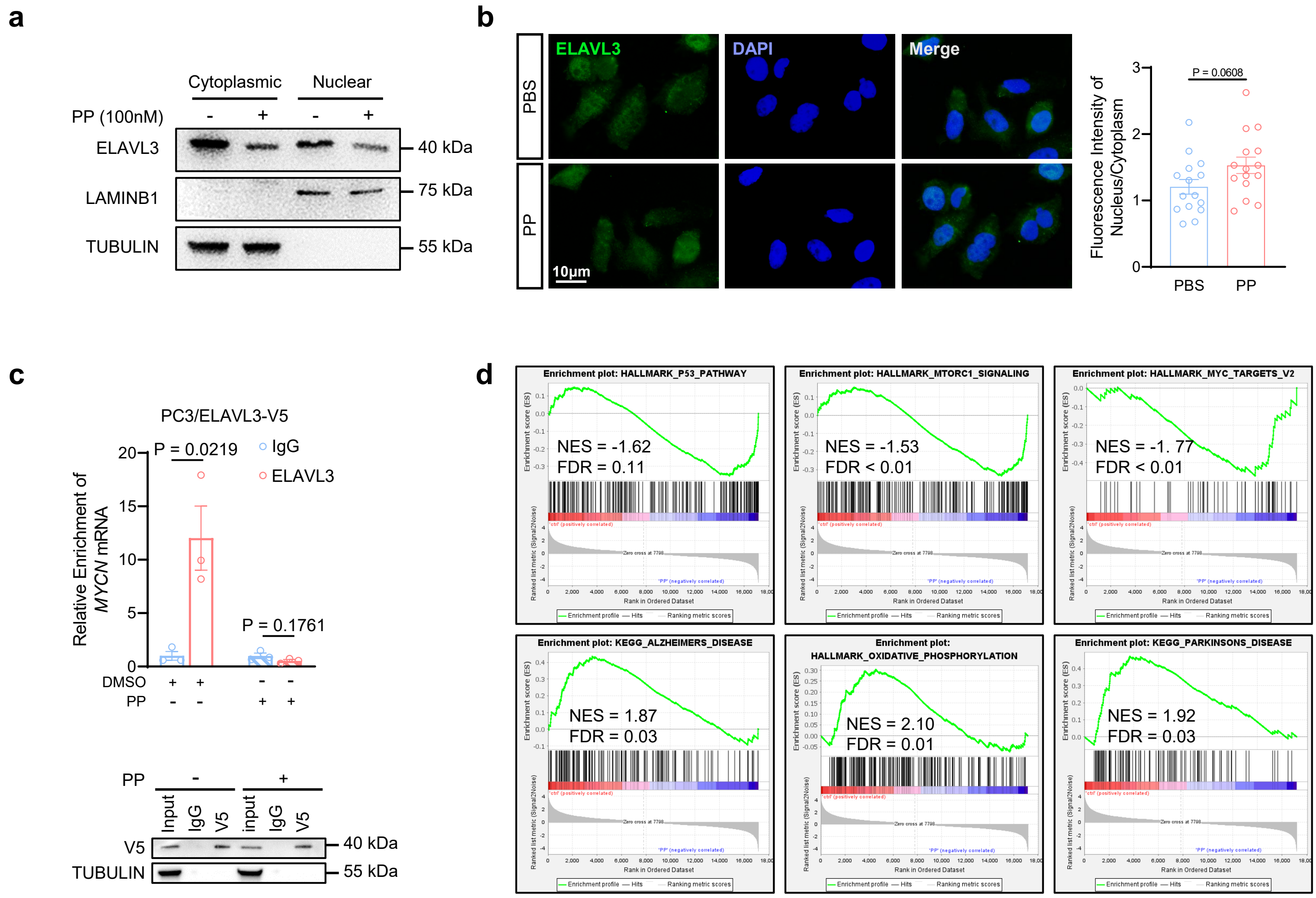

Supplementary Figure S6. Pharmacological inhibition of ELAVL3 blocks neuroendocrine differentiation of prostate cancer cell

a, Western blot showing the cytoplasmic/nuclear expression of ELAVL3, LaminB1, and TUBULIN in LASCPC-01 cells treated with 100 nM PP or PBS. **b**, Immunofluorescence showing subcellular localization of ELAVL3 in LASCPC-01 cells treated with PP or PBS (left, Scale bar, 5μm). Quantification of the fluorescence intensity of nuclear/cytoplasm from LASCPC-01 cells treated with PP or PBS (right). **c**, RIP assay using either ELAVL3 antibody or IgG showing the relative enrichment of *MYCN* mRNA in PC3/ELAVL3 cells treated with 100 nM PP or DMSO (upper, n = 3 biologically independent experiments). Western blot showing ELAVL3 was pulled down by V5 antibody in PC3/ELAVL3 cells treated with PP or PBS (bottom). **d**, Gene set enrichment analysis of differentially expressed genes using hallmark gene sets and KEGG pathways in NCI-H660 cells treated with PP versus PBS. RNA-seq was performed on three independent samples. FDR: false discovery rate. Data presented as mean ± s.e.m. (**c**). Statistical significance was determined by two-tailed unpaired Student's t-test (**c**). Western blot experiments were repeated three times independently, with similar results (**a**,**c**). PP, pyrvinium pamoate. Source data are provided as a Source Data file.

Supplementary Figure S7

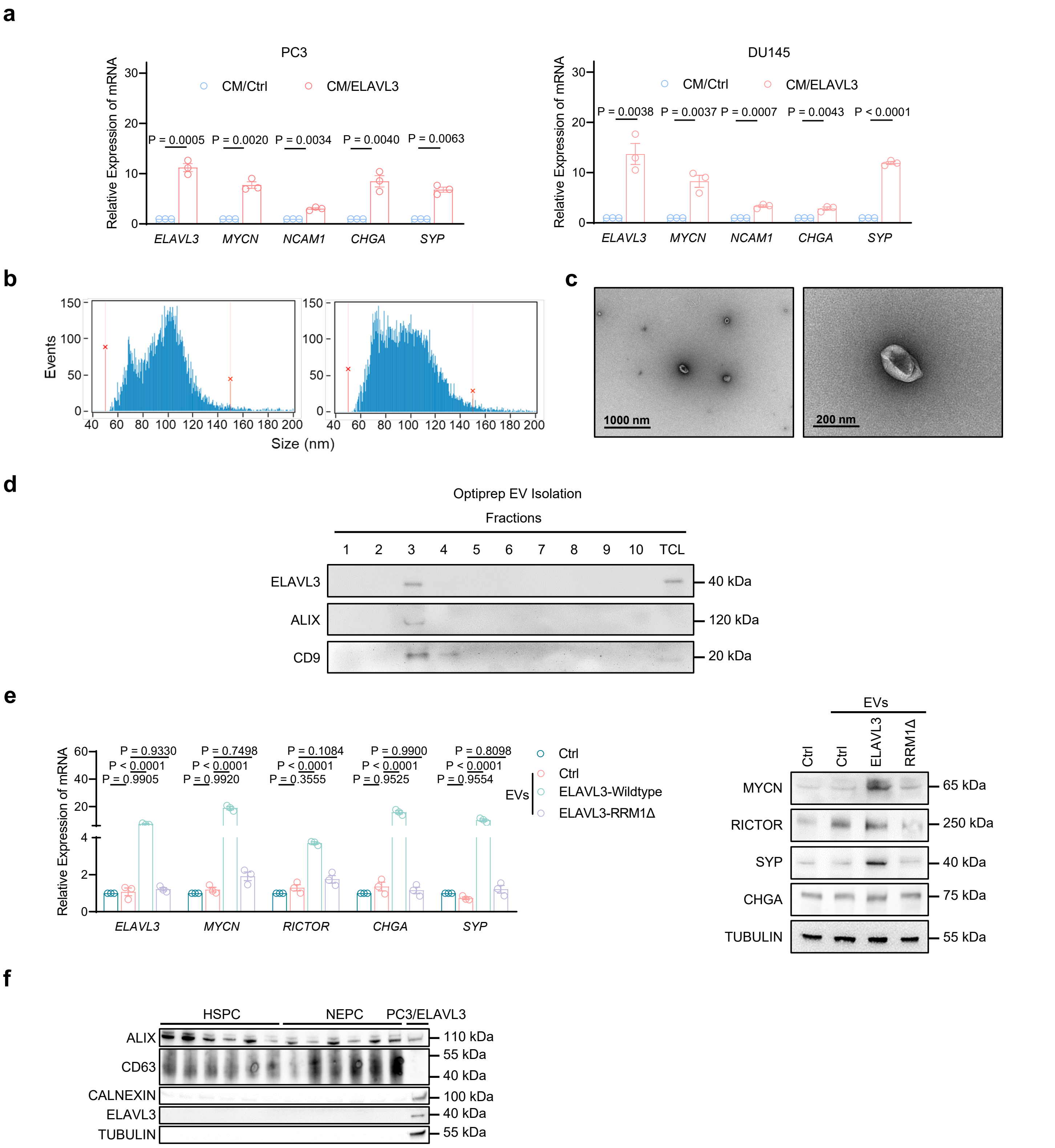

Supplementary Figure S7. Extracellular vesicles-mediated transfer of ELAVL3 induces neuroendocrine differentiation in recipient cancer cells

a, QPCR showing relative mRNA expression of NE-related genes (*MYCN*, *NCAM1*, *CHGA*, and *SYP*) in recipient PC3 (left) and DU145 (right) cells treated with indicated conditioned medium (n = 3 biologically independent experiments). **b**, Flow NanoAnalyzer analysis showing the size range of EVs purified from PC3/Ctrl (left) and PC3/ELAVL3 (right). **c**, Transmission electron microscope showing the morphology of EVs purified from PC3/ELAVL3 cells medium (left, scale bar, 1000 nm; right, scale bar, 200 nm). **d**, Western blot showing ELAVL3, ALIX, and CD9 from different fractions of EVs separated by density gradient with iodixanol/Optiprep. **e**, QPCR showing relative mRNA expression of *ELAVL3*, *RICTOR*, and NE-related genes (*MYCN*, *CHGA*, and *SYP*) in recipient PC3 cells cultured with EVs purified from indicated PC3 cells (left, n = 3 biologically independent experiments). Western blot showing protein expression of RICTOR and NE-related genes (MYCN, SYP, and CHGA) in recipient PC3 cells cultured

with EVs purified from indicated PC3 cells (right). **f**, Western blot showing protein expression of ELAVL3, ALIX, CD63, CALNEXIN, and TUBULIN from PC3/ELAVL3 cell lysate and EVs purified from HSPC (n = 6) and NEPC (n = 6) patients. Data presented as mean ± s.e.m. (**a,e**). Statistical significance was determined by two-tailed unpaired Student's t-test (**a**), or one-way ANOVA with Dunnett's multiple comparisons (**e**). Western blot experiments were repeated three times independently, with similar results (**d,e,f**). HSPC, hormone-sensitive prostate cancer. NEPC, Neuroendocrine prostate cancer. Source data are provided as a Source Data file.

Supplementary Figure S8

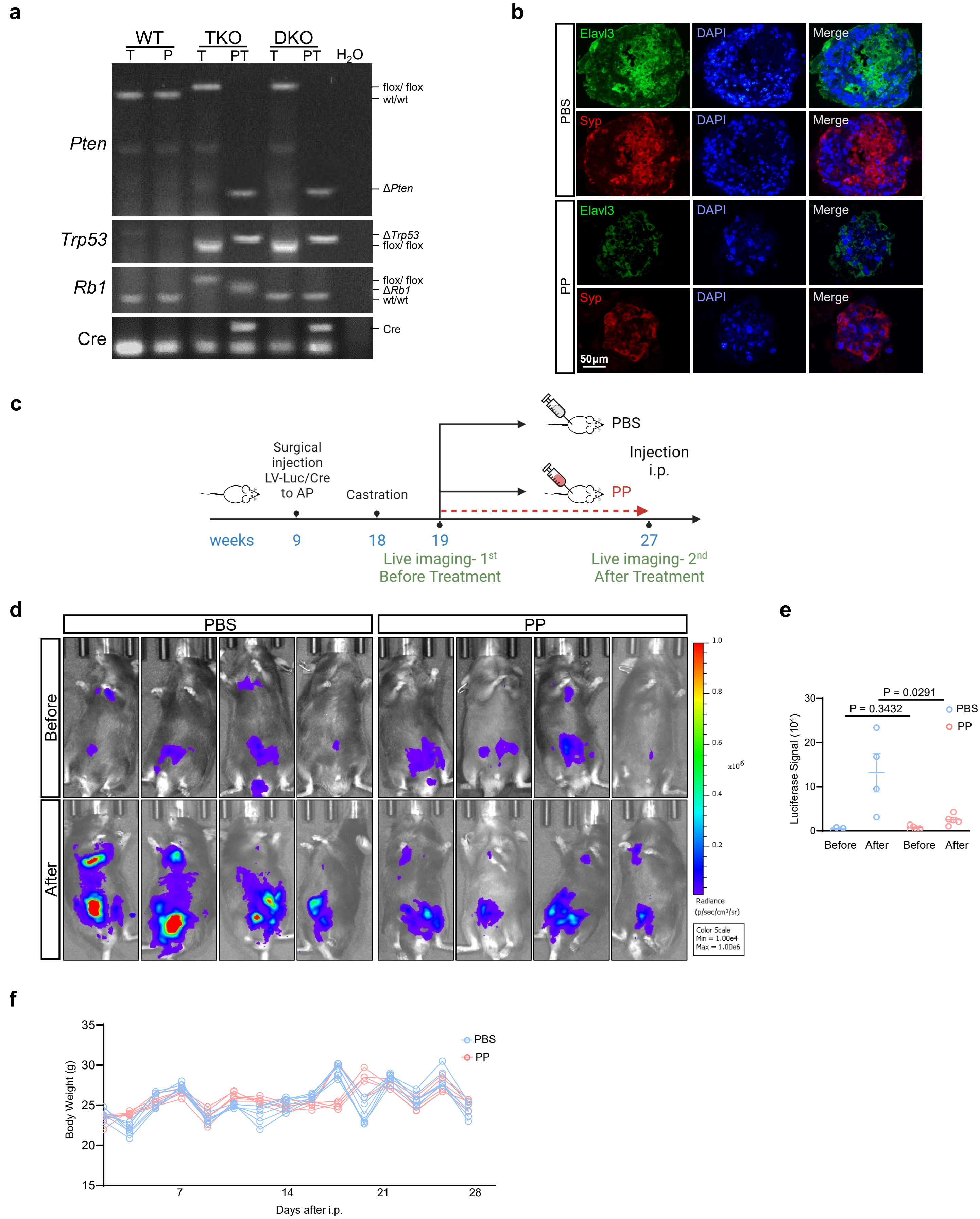

Supplementary Figure S8. Pharmacological inhibition of ELAVL3 suppresses tumor growth and promotes survival in NEPC mice model

a, PCR-based genotyping assays for tissues from tail (T), prostate (P), or prostate tumor (PT) from various genotypes mice. Wildtype (wt), floxed (flox) or Cre recombined alleles (Δ) of the *Pten*, *Trp53*, *Rb1*, or Cre recombined alleles (Δ) were amplified by PCR and visualized by agarose gel electrophoresis. **b**, Representative immunofluorescence staining of Elavl3 and Syp in organoids from

Pb-Cre4: *Pten*$^{f/f}$; *Trp53*$^{f/f}$; *Rb1*$^{f/f}$ prostate tumors treated with PBS or PP. Scale bar, 50 μm. **c**, Schematic illustration showing the treatment and live luciferase imaging of *Pten*$^{f/f}$; *Trp53*$^{f/f}$; *Rb1*$^{f/f}$ mice model with Luc.Cre injection. **d**, Live luciferase imaging of Pb-Cre4: *Pten*$^{f/f}$; *Trp53*$^{f/f}$; *Rb1*$^{f/f}$ mice model with Luc.Cre injection before and after indicated treatment (n = 4 mice per group). **e**, Quantification of the live luciferase signal from Pb-Cre4: *Pten*$^{f/f}$; *Trp53*$^{f/f}$; *Rb1*$^{f/f}$ mice model with Luc.Cre injection before and after indicated treatment. **f**, Changes in body weight of mice treated with PP or vehicle control (n = 4 mice per group). Data presented as mean ± s.e.m. (**e**). Statistical significance was determined by two-tailed paired Student's t-test (**e**). PP, pyrvinium pamoate. Δ, deletion. Source data are provided as a Source Data files.

Supplementary Table 1. Patient cohort in immunohistochemistry staining.

AR status	BPH	HSPC	CRPC	NEPC
No. of patients	15	144	15	22
Age (years)				
<65	4	39	3	5
>=65	11	105	12	17
Gleason Score				
<8		90	2	2
>=8		36	7	8
Non-applicable	15	18	6	12
Clinical T stage				
T1-2	-	94	2	4
T3-4	-	50	13	18
Median serum PSA（ng/mL）	3.8	9.57	37.2	4.33
Sample resource				
Radical surgery		65	2	3
Puncture biopsy	15	79	13	19

Supplementary Table 2. Putative MYCN binding motifs on *ELAVL3* promoter identified by JASPAR.

Predicted MYCN binding sites					
Promoter	Site ID	Start	End	Predicted site sequence	Track Score in JASPAR CORE
ELAVL3	BS1	-1790	-1783	C**CACGTG**A	481
	BS2	-1755	-1748	G**CACGTG**C	424
	BS3	-125	-104	C**CACCTG**C	325

Supplementary Table 3. Putative ELAVL3 binding position to the *MYCN* transcript using the catRAPID algorithm.

Protein ID	RNA ID	Start	End	Ranking
Q14576_ELAVL3	*MYCN* 3'UTR	1	52	0.763292
Q14576_ELAVL3	*MYCN* 3'UTR	26	77	0.762167
Q14576_ELAVL3	*MYCN* 3'UTR	7	58	0.755375
Q14576_ELAVL3	*MYCN* 3'UTR	32	83	0.75525
Q14576_ELAVL3	*MYCN* 3'UTR	51	102	0.753375
Q15717_ELAVL1	*MYCN* 3'UTR	107	158	0.481625
Q15717_ELAVL1	*MYCN* 3'UTR	82	133	0.481083
P26378_ELAVL4	*MYCN* 3'UTR	107	158	0.502042
P26378_ELAVL4	*MYCN* 3'UTR	157	208	0.498875

Supplementary Table 4. Amino acid sequence alignments and RNA recognition motifs for ELAVL3 of indicated species.

```
Danio rerio      ·MVTIISTMETQVSNGPSGTSLPNGPVISTNGATDDSKTNLIVNYLPQNMTQEEFKSLFG
Mus musculus     ·MVTIISTMETQANNGPGCVGILNG····TNGEADDSKTNLIVNYLPQNMTQEEFKSLFG
Xenopus laevis   MVTQILGAMESQVGGGPAGPALPNGPLLGTNGATDDSKTNLIVNYLPQNMTQDEFKSLFG
Homo sapiens     MVTQILGAMESQVGGGPAGPALPNGPLLGTNGATDDSKTNLIVNYLPQNMTQDEFKSLFG
                                              RRM1

Danio rerio      SIGEIESCKLVRDKITGQSLGYGFVNYVDPNDADKAINTLNGLKLQTKTIKVSYARPSSA
Mus musculus     SIGEIESCKLVRDKITGQSLGYGFVNYVDPNDADKAINTLNGLKLQTKTIKVSYARPSSA
Xenopus laevis   SIGDIESCKLVRDKITGQSLGYGFVNYSDPNDADKAINTLNGLKLQTKTIKVSYARPSSA
Homo sapiens     SIGDIESCKLVRDKITGQSLGYGFVNYSDPNDADKAINTLNGLKLQTKTIKVSYARPSSA
                                              RRM1

Danio rerio      SIRDANLYVSGLPKTMSQKDMEQLFSQYGRIITSRILVDQVTAGISRGVGFIRFDKRNEA
Mus musculus     SIRDANLYVSSLPKTMNQKEMEQLFSQYGRIITSRILVDQVTG·VSRGVGFIRFDKRIEA
Xenopus laevis   SIRDANLYVSGLPKTMSQKEMEQLFSQYGRIITSRILLDQATG·VSRGVGFIRFDKRIEA
Homo sapiens     SIRDANLYVSGLPKTMSQKEMEQLFSQYGRIITSRILVDQVTG·VSRGVGFIRFDKRIEA
                                              RRM2

Danio rerio      EEAIKGLNGQKPLGAAEPITVKFANNPSQKTGQALLTQLYQTAARRYTGPLHHQTQRFRL
Mus musculus     EEAIKGLNGQKPLGASEPITVKFANNPSQKTGQALLTHLYQTTARRYTGPLHHQTQRFSP
Xenopus laevis   EEAIKGLNGQKPLGAAEPITVKFANNPSQKTGQALLTHLYQSSARRYAGPLHHQTQRFRL
Homo sapiens     EEAIKGLNGQKPLGAAEPITVKFANNPSQKTGQALLTHLYQSSARRYAGPLHHQTQRFRL
                           RRM2                            Hinge

Danio rerio      DNLLNASY·······GVKRFSPITIDSMTSLAGVNLTG·PTGAGWCIFVYNLSPEADESV
Mus musculus     LSILPR·············FSPITIDSVTNLAGVSLTG·PTTAGWCIFVYNLSPEADESV
Xenopus laevis   DNLLNMAYGVKSPLSLIARFSPIAIDGMSGLAGVGLSGGAAGAGWCIFVYNLSPEADESV
Homo sapiens     DNLLNMAYGVKSPLSLIARFSPIAIDGMSGLAGVGLSGGAAGAGWCIFVYNLSPEADESV
                                   Hinge                          RRM3

Danio rerio      LWQLFGPFGAVTNVKVIRDFTTNKCKGFGFVTMTNYDEAAMAIASLNGYRLGDRVLQVSF
Mus musculus     LWQLFGPFGAVTNVKVIRDFTTNKCKGFGFVTMTNYDEAAMAIASLNGYRLGDRVLQVSF
Xenopus laevis   LWQLFGPFGAVTNVKVIRDFTTNKCKGFGFVTMTNYDEAAMAIASLNGYRLGERVLQVSF
Homo sapiens     LWQLFGPFGAVTNVKVIRDFTTNKCKGFGFVTMTNYDEAAMAIASLNGYRLGERVLQVSF
                                              RRM3

Danio rerio      KTSKQHKA
Mus musculus     KTSKQHKA
Xenopus laevis   KTSKQHKA
Homo sapiens     KTSKQHKA
                 RRM3
```

The homology between species was highlighted in red.

Supplementary Table 5. Putative ELAVL3 binding position to the *RICTOR* transcript using the catRAPID algorithm.

Protein ID	RNA ID	Start	End	Ranking
Q14576_ELAVL3	*RICTOR* 3'UTR	1504	1889	0.732833
Q14576_ELAVL3	*RICTOR* 3'UTR	1500	1885	0.731
Q14576_ELAVL3	*RICTOR* 3'UTR	1120	1505	0.719375
Q14576_ELAVL3	*RICTOR* 3'UTR	2656	3041	0.718167
Q14576_ELAVL3	*RICTOR* 3'UTR	2464	2849	0.717458
Q15717_ELAVL1	*RICTOR* 3'UTR	348	733	0.757208
Q15717_ELAVL1	*RICTOR* 3'UTR	352	737	0.756333
P26378_ELAVL4	*RICTOR* 3'UTR	160	545	0.755333
P26378_ELAVL4	*RICTOR* 3'UTR	156	541	0.755292

Supplementary Table 6. Antibodies used in this study.

Name	Source	Catalog	Host	Dilution	Experiment
N-myc	CST	51705	Rabbit	1:50	ChIP
Rabbit IgG	Abclonal	A005	Rabbit	1:100	ChIP
V5	CST	13202	Rabbit	1:50	RIP
Rabbit IgG	Abclonal	A005	Rabbit	1:100	RIP
ELAVL3	abcam	ab184267	Rabbit	1:100	Immunostaining
CK-8	abcam	ab53280	Rabbit	1:200	Immunostaining
N-myc	abcam	ab198912	Rabbit	1:100	Immunostaining
CHGA	abcam	ab283265	Rabbit	1:2000	Immunostaining
SYP	abcam	ab32127	Rabbit	1:500	Immunostaining
AR	abcam	ab133273	Rabbit	1:100	Immunostaining
Ki67	proteintech	27309-1-AP	Rabbit	1:2000	Immunostaining
NCAM-1	proteintech	14255-1-AP	Rabbit	1:1000	Immunostaining
Cleaved-caspase-3	CST	9661	Rabbit	1:1000	Immunostaining
N-myc	CST	51705	Rabbit	1:1000	western blot
GAPDH	CST	5174	Rabbit	1:1000	western blot
SYP	abcam	ab32127	Rabbit	1:1000	western blot
ENO2	Novus	NB200-421	Mouse	1:1000	western blot
ELAVL3	abcam	ab184267	Rabbit	1:1000	western blot
NCAM1	Proteintech	14255-1-AP	Rabbit	1:1000	western blot
TUBULIN	Abclonal	AC030	Mouse	1:1000	western blot
AKT	CST	4685	Rabbit	1:1000	western blot
p-AKT	CST	4060	Rabbit	1:1000	western blot
S6	Abclonal	A11874	Rabbit	1:1000	western blot
p-S6	Abclonal	AP0538	Rabbit	1:1000	western blot
LAMINB1	CST	17416	Rabbit	1:1000	western blot
V5	CST	13202	Rabbit	1:1000	western blot
RICTOR	abcam	ab70374	Rabbit	1:1000	western blot
PARP1	Abclonal	A19596	Rabbit	1:1000	western blot
cPARP1	Abclonal	A19612	Rabbit	1:1000	western blot
CD63	abcam	ab134045	Rabbit	1:1000	western blot

ALIX	abcam	ab275377	Rabbit	1:1000	western blot
TSG101	abcam	ab125011	Rabbit	1:1000	western blot
CALNEXIN	abcam	ab133615	Rabbit	1:1000	western blot
GAPDH	CST	5174	Rabbit	1:1000	western blot
ELAVL3	Thermo	A-21271	Mouse	1:100	Immunofluoresence
SYP	Santa cruz	5174	Rabbit	1:100	Immunofluoresence

Reference:

1 Ku, S. Y. et al. Rb1 and Trp53 cooperate to suppress prostate cancer lineage plasticity, metastasis, and antiandrogen resistance. Science 355, 78-83, doi:10.1126/science.aah4199 (2017).

2 Beltran, H. et al. Divergent clonal evolution of castration-resistant neuroendocrine prostate cancer. Nat Med 22, 298-305, doi:10.1038/nm.4045 (2016).

3 Cancer Genome Atlas Research, N. The Molecular Taxonomy of Primary Prostate Cancer. Cell 163, 1011-1025, doi:10.1016/j.cell.2015.10.025 (2015).
