# Supplementary material for: The ELAVL3/MYCN positive feedback loop provides a therapeutic target for neuroendocrine prostate cancer
Source: Nat Commun. 2023 Nov 28;14:7794. doi: 10.1038/s41467-023-43676-3 (PMC10684895; doi:10.1038/s41467-023-43676-3)
Supplement: Supplementary file 3 — Description of Additional Supplementary Files [file 41467_2023_43676_MOESM3_ESM.pdf]

Title: Supplementary Data 1:

Description: Primers and oligos used in this study.
