# Supplementary material for: The ELAVL3/MYCN positive feedback loop provides a therapeutic target for neuroendocrine prostate cancer
Source: Nat Commun. 2023 Nov 28;14:7794. doi: 10.1038/s41467-023-43676-3 (PMC10684895; doi:10.1038/s41467-023-43676-3)
Supplement: Supplementary file 5 — Reporting Summary [file 41467_2023_43676_MOESM5_ESM.pdf]

nature portfolio

Corresponding author(s): Qi Wang, Wei Xue
Last updated by author(s): Oct 24, 2023

Reporting Summary

Nature Portfolio wishes to improve the reproducibility of the work that we publish. This form provides structure for consistency and transparency in reporting. For further information on Nature Portfolio policies, see our Editorial Policies and the Editorial Policy Checklist.

Statistics

For all statistical analyses, confirm that the following items are present in the figure legend, table legend, main text, or Methods section.

n/a	Confirmed	
☐	☒	The exact sample size (*n*) for each experimental group/condition, given as a discrete number and unit of measurement
☐	☒	A statement on whether measurements were taken from distinct samples or whether the same sample was measured repeatedly
☐	☒	The statistical test(s) used AND whether they are one- or two-sided<br>*Only common tests should be described solely by name; describe more complex techniques in the Methods section.*
☒	☐	A description of all covariates tested
☒	☐	A description of any assumptions or corrections, such as tests of normality and adjustment for multiple comparisons
☐	☒	A full description of the statistical parameters including central tendency (e.g. means) or other basic estimates (e.g. regression coefficient) AND variation (e.g. standard deviation) or associated estimates of uncertainty (e.g. confidence intervals)
☐	☒	For null hypothesis testing, the test statistic (e.g. *F*, *t*, *r*) with confidence intervals, effect sizes, degrees of freedom and *P* value noted<br>*Give P values as exact values whenever suitable.*
☒	☐	For Bayesian analysis, information on the choice of priors and Markov chain Monte Carlo settings
☒	☐	For hierarchical and complex designs, identification of the appropriate level for tests and full reporting of outcomes
☒	☐	Estimates of effect sizes (e.g. Cohen's *d*, Pearson's *r*), indicating how they were calculated

Our web collection on statistics for biologists contains articles on many of the points above.

Software and code

Policy information about availability of computer code

Data collection

Western blot: ECL detection system (Tanon, China)(version 5200 Multi).
Quantitative PCR: LightCycler 480 qPCR machine (Roche)(version 480 II).
RNA-sequencing: RNA integrity was measured and the library was quantified using Agilent 2100 Bioanalyzer (Agilent, Santa Clara, CA). Sequencing was performed on the Illumina HiSeq2000 system (Illumina, San Diego, CA) at high out-put model according to the manufacturer's instruction.
RIP-sequencing:RNA integrity was measured and the library was quantified using Agilent 2100 Bioanalyzer (Agilent, Santa Clara, CA). Sequencing was performed on the Illumina HiSeq2000 system (Illumina, San Diego, CA) at high out-put model according to the manufacturer's instruction.
PET/CT imaging: PET/CT imaging was performed on an Inveon MM Platform (Siemens Preclinical Solutions, Knoxville, Tennessee, USA)
Bioluminescence imaging: In vivo bioluminescence imaging was performed using a Xenogen IVIS Spectrum imager.
Transmission electron microscopy: Transmission electron microscope (HT7800, HITACHI) at 80kV.
Cell counting kit-8 assay: The absorbance values at 450 nm were measured with Thermo Fisher Scientific SkanIt Software 4.1.

Data analysis

RIP-sequencing: Raw reads were filtered with Fastp (v0.20.0), an ultra-fast all-in-one FASTQ preprocessor, to obtain high-quality clean reads by removing sequencing adapters, short reads (length < 30 bp) and low-quality reads. Then FastQC was employed to ensure high reads quality and to process data quality control. Differentially expressed genes between two groups were calculated in R v4.0.5 by limma package v3.46.0. P < 0.05 with log2FoldChange > 1 or < -1 was set as cutoff. Gene set enrichment analysis of the differentially expressed genes was performed on the web-based Metascpae via www.metascape.org.
RNA-sequencing: Raw data were filtered by removing reads of low quality (over 10% uncertain bases or over 50% bases of Q < 5) or containing adapters, and approximately 20 million clean reads per sample were sampled for following analysis. Transcriptome reads were mapped to the

reference genome (hg19) using the Bowtie tool. Gene expression level was quantified using RSEM software. Differentially expressed genes between two groups were calculated in R v4.0.5 by limma package v3.46.0. P < 0.001 with log2FoldChange > 1 or < -1 was set as cutoff. Gene set enrichment analysis was performed on GSEA function of clusterProfiler package v3.18.1 or GSEA software v4.1.0 to analyze the biological difference.
Single-cell-RNA-sequence reanalysis: The raw gene expression matrix of single-cell transcriptome was derived from GSE137829 and further analyzed in R 4.0.5 using Seurat package v4.0.2.
[18F]-FDG PET/CT: Images were reconstructed by an OSEM3D (Three-Dimensional Ordered Subsets Expectation Maximum) algorithm followed by MAP (Maximization/Maximum a Posteriori) or FastMAP provided by IAW. The 3D regions of interest (ROIs) were drawn over the heart guided by CT images and tracer uptake was measured using the software of Inveon Research Workplace (IRW) 3.0. Individual quantification of the [18F]-FDG uptake in each of them was calculated.
Bioluminescence Imaging: The measured signal was quantified as photons/second (ph/sec) using the Living Image software v4.2 (Xenogen).
Nanoflow cytometry analysis: Relevant size and concentration were acquired after the conversion of the flow rate and side-scattering intensity of granules based on calibration curve on the NanoFCM software (NanoFCM Profession V2.0).
All statistical analyses were performed with GraphPad 8.0 software.

For manuscripts utilizing custom algorithms or software that are central to the research but not yet described in published literature, software must be made available to editors and reviewers. We strongly encourage code deposition in a community repository (e.g. GitHub). See the Nature Portfolio guidelines for submitting code & software for further information.

Data

Policy information about availability of data

All manuscripts must include a data availability statement. This statement should provide the following information, where applicable:

- Accession codes, unique identifiers, or web links for publicly available datasets
- A description of any restrictions on data availability
- For clinical datasets or third party data, please ensure that the statement adheres to our policy

The RIP-seq and RNA-seq data generated in this study have been deposited in the NCBI database under accession code GSE224911 [https://www.ncbi.nlm.nih.gov/geo/query/acc.cgi?acc=GSE224911]. The publicly available prostate cancer clinical data, RNA-seq data, and ChIP-seq data used in this study are available in the cBioPortal database (www.cbioportal.org) and the GEO database under accession code: GSE90891, GSE86532, GSE137829, and GSE1173064. The remaining data are available within the Article, Supplementary Information or Source Data file. Source data are provided with this paper.

Research involving human participants, their data, or biological material

Policy information about studies with human participants or human data. See also policy information about sex, gender (identity/presentation), and sexual orientation and race, ethnicity and racism.

Reporting on sex and gender	Participants were Chinese male, aged from 57-82.
Reporting on race, ethnicity, or other socially relevant groupings	N/A
Population characteristics	A total of 15 BPH(average age:66 years), 144 HSPC (average age:67 years), 15 CRPC (average age:70 years), 22 NEPC (average age:66 years) patient samples were obtained from Ren Ji Hospital (SJTU, Shanghai, China) between 2008 and 2021.
Recruitment	Prostate cancer patients were recruited by Ren Ji Hospital, Shanghai Jiao Tong University School of Medicine, China. There is no potential bias for recruiting patient cohort. All samples were anonymous.
Ethics oversight	The collection of human samples and research conducted in this study was approved by the Research Ethics Committee of the Renji Hospital, Shanghai Jiao Tong University School of Medicine (approval numbers: KY2022-136A). Clinical samples and information were collected after written informed consent.

Note that full information on the approval of the study protocol must also be provided in the manuscript.

Field-specific reporting

Please select the one below that is the best fit for your research. If you are not sure, read the appropriate sections before making your selection.

[x] Life sciences   [ ] Behavioural & social sciences   [ ] Ecological, evolutionary & environmental sciences

For a reference copy of the document with all sections, see nature.com/documents/nr-reporting-summary-flat.pdf

Life sciences study design

All studies must disclose on these points even when the disclosure is negative.

Sample size

Sample sizes were established based on data from preliminary experiments and findings from related studies to ensure robust statistical analysis (Davies et al, Nature Cell Biology, 2021; Bishop et al, Cancer Discovery, 2017). For in vitro experiments, at least three biologically independent experiments were performed for all experiments unless otherwise stated. No statistical method was used to predetermine sample size. Such sample sizes are typical for the in vitro experiments and sufficient for a statistical analysis. For in vivo experiments, a sample size of n = 5-6 mice per group were used, which is sufficient to generate statistically significant results. No statistical method was used to

	predetermine sample size. For patients sample size, no prespecified samples sizes were determined. The sample size resulted from profiling/ analyzing all prostate cancer samples available to the research team, and the size of patients are sufficient for a statistical analysis.
Data exclusions	No data were excluded throughout the studies
Replication	The replication numbers were described in the corresponding figure legends.
Randomization	For in vitro experiments, cells were randomly allocated into control and experimental groups. For in vivo experiments, age matched mice were randomized into all experimental groups.
Blinding	Data collection of mouse tumor experiments were performed in a double blinding manner, the investigators were blinded to group allocation during "data analysis' of mouse tumor experiments, Cellular and biochemical experiments were not performed in a blinding manner because the same investigator was doing group allocation during data collection and/or analysis. For other experiments, investigators were not blinded to the identity of samples to ensure appropriate data collection and because experimental results are quantitative in nature, not readily subject to investigator bias. To ensure consistent experimental conditions, all control and experimental samples were processed in parallel.

Reporting for specific materials, systems and methods

We require information from authors about some types of materials, experimental systems and methods used in many studies. Here, indicate whether each material, system or method listed is relevant to your study. If you are not sure if a list item applies to your research, read the appropriate section before selecting a response.

Materials & experimental systems

n/a	Involved in the study
☐	☒ Antibodies
☐	☒ Eukaryotic cell lines
☒	☐ Palaeontology and archaeology
☐	☒ Animals and other organisms
☒	☐ Clinical data
☒	☐ Dual use research of concern
☒	☐ Plants

Methods

n/a	Involved in the study
☒	☐ ChIP-seq
☒	☐ Flow cytometry
☒	☐ MRI-based neuroimaging

Antibodies

Antibodies used

N-myc (CST, 51705, 1:50, ChIP),
Rabbit IgG (Abclonal, AC005,1:100, ChIP),
V5 (CST,13202, 1:50, RIP),
Rabbit IgG (Abclonal , A005 , 1:100 , RIP),
ELAVL3 (abcam , ab184267 , 1:100 , Immunostaining),
CK-8 (abcam , ab53280 , 1:200 , Immunostaining),
N-myc (abcam , ab198912 , 1:100 , Immunostaining),
CHGA (abcam , ab283265 , 1:2000 , Immunostaining),
SYP (abcam , ab32127 , 1:500 , Immunostaining),
AR (abcam , ab133273 , 1:100 , Immunostaining),
Ki67 (proteintech , 27309-1-AP , 1:2000 , Immunostaining),
NCAM-1 (proteintech , 14255-1-AP , 1:1000 , Immunostaining),
Cleaved-caspase-3 (CST , 9661 , 1:1000 , Immunostaining),
N-myc (CST , 51705 , 1:1000 , Western blot),
GAPDH (CST , 5174 , 1:1000 , Western blot),
SYP (abcam , ab32127 , 1:1000 , Western blot),
ENO2 (Novus , NB200-421 , 1:1000 , Western blot),
ELAVL3 (abcam , ab184267 , 1:1000 , Western blot),
NCAM1 (Proteintech , 14255-1-AP , 1:1000 , Western blot),
TUBULIN (Abclonal , AC030 , 1:1000 , Western blot),
AKT (CST , 4685 , 1:1000 , Western blot),
p-AKT (CST , 4060 , 1:1000 , Western blot),
S6 (Abclonal , A11874 , 1:1000 , Western blot),
p-S6 (Abclonal , AP0538 , 1:1000 , Western blot),
LAMINB1 (CST , 17416 , 1:1000 , Western blot),
V5 (CST , 13202 , 1:1000 , Western blot),
RICTOR (abcam , ab70374 , 1:1000 , Western blot),
PARP1 (Abclonal , A19596 , 1:1000 , Western blot),
cPARP1 (Abclonal , A19612 , 1:1000 , Western blot),
CD63 (abcam , ab134045 , 1:1000 , Western blot),
ALIX (abcam , ab275377 , 1:1000 , Western blot),

TSG101 (abcam , ab125011 , 1:1000 , Western blot),
CALNEXIN (abcam , ab133615 , 1:1000 , Western blot),
GAPDH (CST , 5174 , 1:1000 , Western blot),
ELAVL3 (Thermo , A-21271 , 1:100 , Immunofluoresence),
SYP (Santa cruz , 5174 , 1:100 , Immunofluoresence).

Validation

Rabbit monoclonal anti-N-myc was validated for ChIP in a human cell line. https://www.cellsignal.cn/products/primary-antibodies/n-myc-d4b2y-rabbit-mab/51705
Rabbit anti-IgG was validated for ChIP in a human cell line. https://abclonal.com.cn/catalog/AC005
Rabbit monoclonal anti-V5 was validated for RIP in a human cell line. https://www.cellsignal.cn/products/primary-antibodies/v5-tag-d3h8q-rabbit-mab/13202
Rabbit anti-IgG was validated for RIP in a human cell line. https://abclonal.com.cn/catalog/AC005
Rabbit monoclonal anti-ELAVL3 was validated for immunostaining in human samples. https://www.abcam.cn/products/primary-antibodies/hud--huc-antibody-epr19098-ab184267.html
Rabbit monoclonal anti-CK8 was validated for immunostaining in human samples. https://www.abcam.cn/products/primary-antibodies/cytokeratin-8-antibody-ep1628y-cytoskeleton-marker-ab53280.html
Rabbit polyclonal anti-MYCN was validated for immunostaining in human samples. https://www.abcam.cn/products/primary-antibodies/n-mycmycn-antibody-ab198912.html
Rabbit recombinant multiclonal anti-CHGA was validated for immunostaining in human samples. https://www.abcam.cn/products/primary-antibodies/chromogranin-a-antibody-rm1025-ab283265.html
Rabbit monoclonal anti-SYP was validated for immunostaining in human samples. https://www.abcam.cn/products/primary-antibodies/synaptophysin-antibody-ye269-ab32127.html
Rabbit monoclonal anti-AR was validated for immunostaining in human samples. https://www.abcam.cn/products/primary-antibodies/androgen-receptor-antibody-epr15352-ab133273.html
Rabbit polyclonal anti-NCAM1 was validated for immunostaining in human samples. https://www.ptgcn.com/products/NCAM1-Antibody-14255-1-AP.htm
Rabbit monoclonal anti-Cleaved-caspase-3 was validated for immunostaining in human samples. https://www.cellsignal.cn/products/primary-antibodies/cleaved-caspase-3-asp175-antibody/9661
Rabbit monoclonal anti-NMYC was validated for immunoblotting in a human cell line. https://www.cellsignal.cn/products/primary-antibodies/n-myc-d4b2y-rabbit-mab/51705
Rabbit monoclonal anti-SYP was validated for immunoblotting in a human cell line. https://www.abcam.cn/products/primary-antibodies/synaptophysin-antibody-ye269-ab32127.html
Rabbit ploypclonal anti-ENO2 was validated for immunoblotting in a human cell line. https://www.novusbio.com/products/enolase-2-neuron-specific-enolase-antibody_nb110-58870
Rabbit monoclonal anti-ELAVL3 was validated for immunoblotting in a human cell line. https://www.abcam.cn/products/primary-antibodies/hud--huc-antibody-epr19098-ab184267.html
Rabbit polyclonal anti-NCAM1 was validated for immunoblotting in a human cell line. https://www.ptgcn.com/products/NCAM1-Antibody-14255-1-AP.htm
Mouse monoclonal anti-Tubulin was validated for immunoblotting in a human cell line. https://abclonal.com.cn/catalog/AC030
Rabbit monoclonal anti-AKT(pan) was validated for immunoblotting in a human cell line. https://www.cellsignal.cn/products/primary-antibodies/akt-pan-11e7-rabbit-mab/4685
Rabbit monoclonal anti-phospho-Akt (Ser473) was validated for immunoblotting in a human cell line. https://www.cellsignal.cn/products/primary-antibodies/phospho-akt-ser473-d9e-xp-rabbit-mab/4060
Rabbit monoclonal anti-RPS6 was validated for immunoblotting in a human cell line. https://abclonal.com.cn/catalog/A11874
Rabbit monoclonal phospho-S6 was validated for immunoblotting in a human cell line. https://abclonal.com.cn/catalog/AP0538
Rabbit monoclonal anti-LAMINB1 was validated for immunoblotting in a human cell line. https://www.cellsignal.cn/products/primary-antibodies/lamin-b1-e6m5t-rabbit-mab/17416
Rabbit monoclonal anti-PARP1 was validated for immunoblotting in a human cell line. https://abclonal.com.cn/catalog/A19596
Rabbit monoclonal anti-cleaved-PARP1 was validated for immunoblotting in a human cell line. https://abclonal.com.cn/catalog/A19612
Rabbit monoclonal anti-CD63 was validated for immunoblotting in a human cell line. https://www.abcam.cn/products/primary-antibodies/cd63-antibody-epr5702-late-endosome-marker-ab134045.html
Rabbit monoclonal anti-ALIX was validated for immunoblotting in a human cell line. https://www.abcam.cn/products/primary-antibodies/alix-antibody-epr23653-32-ab275377.html
Rabbit monoclonal anti-TSG101 was validated for immunoblotting in a human cell line. https://www.abcam.cn/products/primary-antibodies/tsg101-antibody-epr7130b-ab125011.html
Rabbit monoclonal anti-Calnexin was validated for immunoblotting in a human cell line. https://www.abcam.cn/products/primary-antibodies/calnexin-antibody-epr36332-er-membrane-marker-ab133615.html
Rabbit monoclonal anti-GAPDH was validated for immunofluoresence in a human cell line. https://www.cellsignal.cn/products/primary-antibodies/gapdh-d16h11-xp-rabbit-mab/5174
Mouse monoclonal anti-ELAVL3 was validated for immunofluoresence in a human cell line. https://www.thermofisher.cn/cn/zh/antibody/product/HuC-HuD-Antibody-clone-16A11-Monoclonal/A-21271
Mouse monoclonal anti-SYP was validated for immunofluoresence in a human cell line. https://www.scbt.com/zh/p/syp-antibody-d-4

Eukaryotic cell lines

Policy information about [cell lines and Sex and Gender in Research]

Cell line source(s)

The human prostate cancer cell lines LNCaP (SCSP-5021), 22Rv1 (SCSP-5022), PC3 (SCSP-532), DU145 (SCSP-5024), and VCaP (SCSP-5034)cells were purchased from the Cell Bank, Shanghai Institutes for Biological Sciences, Chinese Academy of

	Sciences, the LAPC4 cell line was provided by Charles Sawyers (Memorial Sloan Kettering Cancer Center, New York, NY) (PMID:10086382), LASCPC-01 (CRL-3356) and NCI-H660 (CRL-5813) cells were purchased from ATCC.
Authentication	Human lines were analyzed with STR profiling by he Cell Bank, Shanghai Institutes for Biological Sciences, Chinese Academy of Sciences and ATCC (before ordering from them), to confirm correct donor our species.
Mycoplasma contamination	All cell lines routinely tested negative for mycoplasma.
Commonly misidentified lines (See ICLAC register)	No commonly misidentified cell lines were used.

Animals and other research organisms

Policy information about studies involving animals; ARRIVE guidelines recommended for reporting animal research, and Sex and Gender in Research

Laboratory animals	6-week-old male BALB/c nude mice were provided by the animal laboratory of Ren Ji Hospital. 4-week-old male Pb-Cre4 (#026662), Trp53f/f (#008462), Rb1f/f (#026563), and Ptenf/f mice (#006440) on the C57BL/6 genetic background were bought from Jackson Laborarory to generate Pb-Cre4: Ptenf/f; Trp53f/f; Rb1f/f mice, Pb-Cre4: Ptenf/f; Trp53f/f mice, and Ptenf/f; Trp53f/f; Rb1f/f mice. Room temperature was maintained at 21–22°C and the humidity at 25–47%. Animals were kept at a 12 h/12 h dark/light cycle and maintained in ventilated cages (4 mice per cage).
Wild animals	No wild animal involve.
Reporting on sex	All animals involved in this study were male.
Field-collected samples	The study did not involve field-collected samples.
Ethics oversight	All animal experiments in the current study were performed according to the ethical regulations of Ren ji Hospital. Animal experiment protocols were approved by the Ren Ji Hospital Laboratory Animal Use and Care Committee.

Note that full information on the approval of the study protocol must also be provided in the manuscript.
